# Neuroborreliosis with involvement of rhombencephalon: A case report

**DOI:** 10.1016/j.idcr.2022.e01472

**Published:** 2022-03-08

**Authors:** Hilde Svingen, Jon Orrem, Arne Nørgaard Eskesen

**Affiliations:** a Department of Internal Medicine, Akershus University Hospital, Mailbox 1000, 1478 Lørenskog, Norway; b Department of Radiology, Akershus University Hospital, Mailbox 1000, 1478 Lørenskog, Norway; c Department of Infectious Diseases, Akershus University Hospital, Mailbox 1000, 1478 Lørenskog, Norway

**Keywords:** ADC, apparent diffusion coefficient, AMPA, aminomethylphosphonic acid, BBB, blood-brain barrier, COR, coronal, CRMP5, collapsing response mediator protein 5, CSF, cerebrospinal fluid, DNER, Delta and Notch-like epidermal growth factor-related, DPPX, dipeptidyl-peptidase-like protein, DWI, diffusion-weighted imaging, FLAIR, fluid-attenuated inversion recovery, GABA, gamma-aminobutyric acid, GAD65, glutamic acid decarboxylase 65-kilodalton isoform, JC polyomavirus, John Cunningham polyomavirus, MRI, Magnetic resonance imaging, NMDA-receptor, N-methyl-D-aspartate receptor, PKCγ, protein kinase C gamma, SOX1, sex-determining region Y-related high mobility group box 1, SAG, sagittal, SPIR, Spectral Presaturation with Inversion Recovery, PCR, polymerase chain reaction, CT, computed tomography, EEG, electroencephalography, Borrelia rhombencephalitis, Neuroborreliosis, *Borrelia burgdorferi* sensu lato, Lyme disease

## Abstract

We describe a case of a 52 year-old woman who was hospitalized with rhombencephalitis caused by *Borrelia burgdorferi* sensu lato. The patient presented with intermittent fever, dry cough, fatigue, global headache, night sweats, unintentional weight loss, and neurological symptoms like diplopia, tremor, paresthesia and ataxia. Examination of serum and cerebrospinal fluid (CSF) revealed positive *Borrelia burgdorferi*-specific antibody index and presence of CSF oligoclonal IgG bands, indicating intrathecal synthesis of *Borrelia-*specific antibodies. The clinical and biochemical picture thus suggested neuroborreliosis. Unexpectedly a magnetic resonance imaging (MRI) scan demonstrated inflammation in rhombencephalon that are extremely rare in patients with neuroborreliosis. The patient was treated with intravenous ceftriaxone with rapid improvement of her symptoms. The MRI findings were in regress six weeks after onset of antibiotic treatment, and normalized after about seven months.

## Highlights

• Rhombencephalitis refers to inflammatory diseases of the rhombencephalon that include the pons, cerebellum and medulla oblongata.
• *Borrelia burgdorferi* sensu lato includes the pathogenic genospecies that causes Lyme disease and is transmitted to humans through the bite of infected ticks in the Ixodidae family, in Europe *Ixodes ricinus*.
• To diagnose neuroborreliosis can be challenging; it can cause a wide range of unspecific symptoms, the serological tests can be difficult to interpret, the initial CSF findings can be unspecific, and direct test methods like PCR have low sensitivity.
• Neuroborreliosis should be considered in all patients with neurological symptoms and findings suggesting inflammation in the CNS, also if normal imaging.
• If the clinical picture suggests neuroborreliosis, initiate treatment as soon as possible, and simultaneously continue to investigate for differential diagnoses if indicated.

## Introduction

Lyme disease is a tick-borne infection caused by the spirochete *Borrelia burgdorferi* sensu lato complex, transmitted to humans through the bite of infected ticks in the family Ixodidae (hard ticks), in Europe mainly *Ixodes ricinus* [1], [2]**.** There are several pathogenic *Borrelia burgdorferi* genospecies and the most common in Europe are *Borrelia afzelii*, *Borrelia garinii* and *Borrelia burgdoferi* sensu stricto, while the latter is the principal etiologic agent in the United States [1], [2].

Early infection causes the readily recognizable skin lesion, erythema migrans, which is by far the most common clinical presentation [3]. Left untreated, infection can disseminate from the site of inoculation to various organs, including the nervous system in approximately 10–15% of patients in both Europe and the United States [3], [4]. Among the *Borrelia burgdorferi* genospecies, *Borrelia garinii* is most strongly associated with neuroborreliosis in Europe [1], [5], [6]. Arthritis is more frequent in patients in the United States compared to Europe, while the range and frequency of neurological manifestations seem to be quite similar [4], [7].

The most common clinical picture of Lyme neuroborreliosis are painful radiculitis, meningitis with lymfocytic pleocytosis and cranial neuritis most often affecting the facial nerve (Bannwarth syndrome), usually presenting within weeks to a few months after the tick bite [8], [9], [10]. Less common is involvement of the central nervous system, with paresis due to myelitis as the most common manifestation [8], [9], [10]. Parenchymal brain involvement resulting in encephalitis is exceedingly rare, estimated to occur in 0,1% of untreated infections [11]. No specific form of encephalitis caused by Lyme disease can be identified from the few cases that exist, but a pattern involving rhombencephalon has been suggested [12]. Rhombencephalitis refers to inflammatory diseases affecting the rhombencephalon that include the pons, cerebellum and medulla oblongata [13].

To diagnose borreliosis can be challenging due to varying and nonspecific symptoms, the serological tests can be difficult to interpret, and imaging findings are often non-specific. In addition, many of the symptoms are common in other diseases, like headache, fatigue and cognitive impairment. The sensitivity of serological tests in early stage of infection are low, and specific IgM and IgG antibodies can remain positive several years after infection, making it difficult to differentiate previous exposure, reinfection and acute infection [12]. Direct test methods for detection of *Borrelia burgdorferi* sensu lato generally have low sensitivity. Use of polymerase chain reaction (PCR) in CSF have a sensitivity of just 19%, regardless of method, DNA target and stage of disease [1]. The sensitivity of PCR in blood or plasma is less than 40%, depending on the clinical picture [14].

Although borreliosis usually is a benign and self-limited infection, some patients can without proper treatment develop late stage disseminated disease. Most patients respond well to antibiotics, but severe illness and late onset of proper treatment increase the risk of complications, including persistent symptoms [12], [15], [16], [17].

Only very few cases in the literature describe neuroborreliosis with involvement of rhombencephalon [18], [19]. We report a case of rhombencephalitis as a manifestation of neuroborreliosis in a 52 year-old woman. MRI of the brain was initially interpreted as suggestive of malignancy or inflammatory disease.

## Case report

A 52 year-old woman was hospitalized with an eight months history with intermittent fever, dry cough, fatigue, global headache, night sweats, unintentional weight loss of about fifteen pounds, binocular diplopia, tremor in the neck, paresthesia and tremor in the extremities, and unsteady gait. The family also noted increasing memory loss. The symptoms had progressively worsening, and at time of admission she was not able to work due to exhaustion. The patient had been exposed to ticks on a holiday in a Lyme-endemic area in Eastern Norway (Holmsbu) about four weeks prior to the onset of symptoms, but did not identify a tick bite or erythema migrans.

Early in the course of symptoms she was seen in both the outpatient Infectious Disease Clinic and the outpatient Neurological Disease Clinic. Due to prolonged symptoms she was referred to computed tomography (CT) imaging of her brain, neck, chest, abdomen and pelvis, and all these examinations were normal except an incidental finding of a myoma of the uterus. She was also referred to an MRI scan of her brain and medulla with normal findings except modest degeneration in the vertebral column. Lumbar puncture was planned, but was postponed due to the Covid-19 pandemic. No diagnosis was established.

On initial presentation at admission she was afebrile, hypertensive to 158/108 mmHg and had tachycardia to 104 beats per minute. Physical examination was unremarkable, including no neck stiffness. Neurological examination noted paresthesia and decreased sensation in the extremities, resting and intentional tremor, ataxia with disturbances in coarse coordination and unsteady gait. During hospitalization she also developed bilateral spasticity, especially in the lower extremities.

Initial laboratory investigation showed results within normal limits of white blood cell count, platelet count, hemoglobin and hematocrit. Her serum electrolytes was unremarkable, she had normal renal and liver function parameters, normal C-reactive protein, thyroid function parameters, normal troponin and creatine kinase.

A new MRI scan of her brain and medulla were performed, this time both without and with gadolinium contrast agent. The MRI now demonstrated pathology in rhombencephalon with quite symmetric T2 hyperintensity involving capsula interna bilaterally, extending through mesencephalon to pons. There was no sign of enhancement on post-contrast images. The findings were suggestive of inflammatory etiology, although malignancy, like lymphoma, could not be excluded, and it was explicitly specified on the radiology report that these findings were extremely rare to see in patients with neuroborreliosis.

**Fig. 1:**
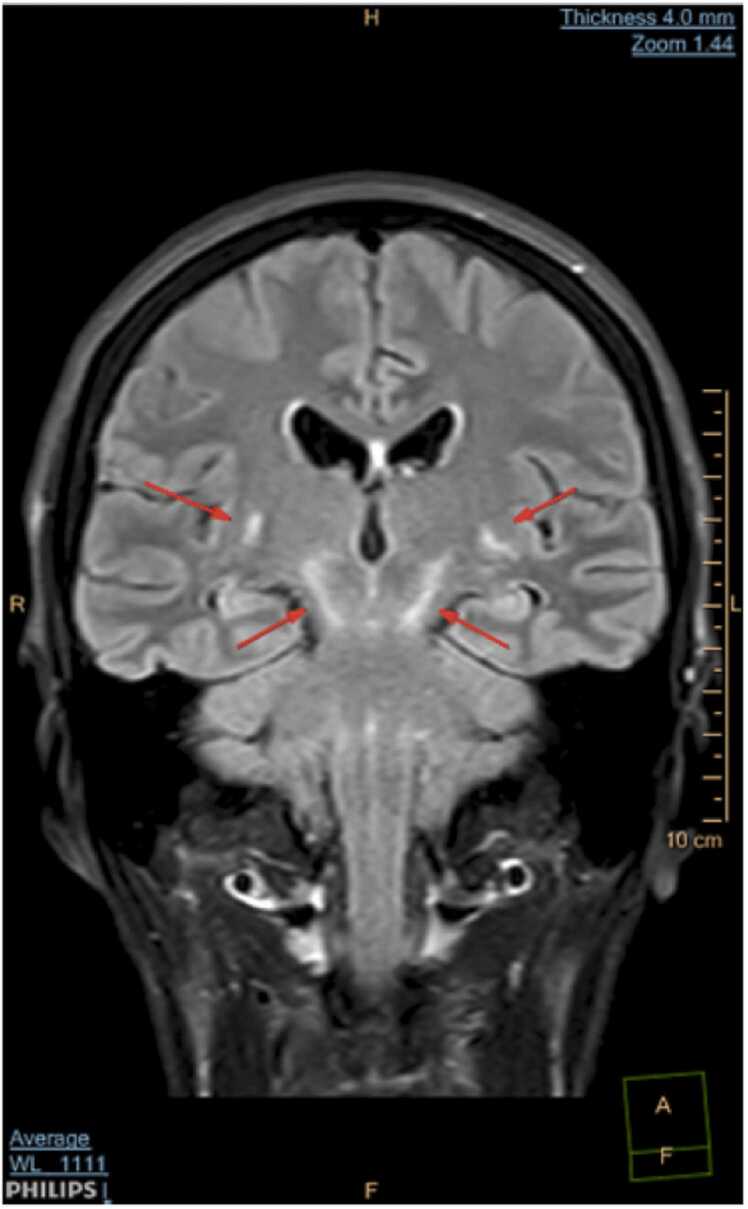
COR T2 FLAIR SPIR.

**Fig. 2:**
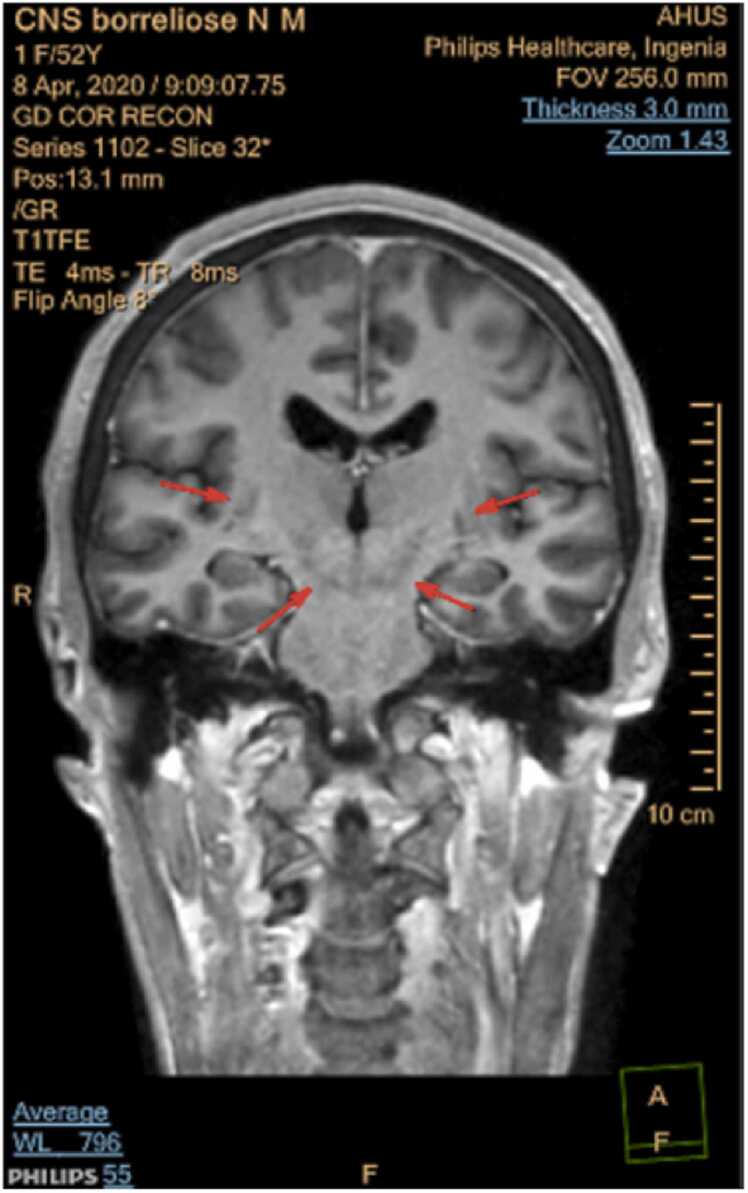
COR T1GD.

**Fig. 3:**
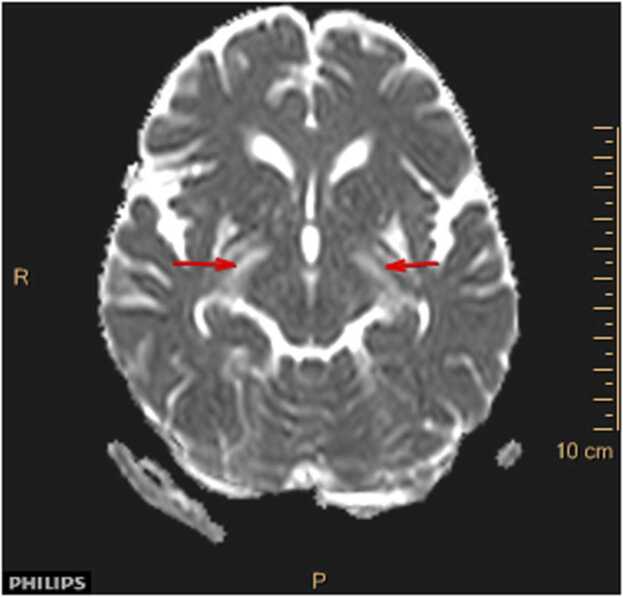
ADC map.

*For imaging we used a Phillips Ingenia 1,5 T. The sequences obtained were DWI with ADC mapping, TRA T2, coronal and sagittal T2 FLAIR SPIR, TRA T1 and 3DT1GD (contrast enhanced). Symmetric T2 hyperintensities radiating from capsula interna through mecencephalon to pons (* Fig. 1 *) with no contrast enhancement (* Fig. 2 *) and normal ADC (no diffusion restriction) (* Fig. 3 *) suggestive of vasogenic edema.*

Electroencephalography (EEG) showed sporadically diffuse polymorph and some theta activity without sharp potentials, considered non-specific, but could also represent generalized dysfunction.

Malignancy was considered to be more unlikely, due to the long duration of symptoms, no signs of malignancy on her previous full body CT-scan, and negative screening for paraneoplastic antibodies in serum (anti-Hu, anti-Ri, anti-Yo, anti-Amphiphysin, anti-CRMP5, anti-Ma1, anti-Ma2, anti-SOX1, anti-Tr(DNER), anti-GAD65, anti-Zic4, anti-PKCγ, anti-Recoverin).

*Borrelia burgdorferi* IgG antibody screening by immunoassay in serum shortly prior to admission was positive, with significant titer rising from 179 AU/mL to 789 AU/mL in about five months. *Borrelia burgdorferi* IgM antibody screening in serum was negative. Simultaneously as obtaining samples of serum at admission, a lumbar puncture was performed. CSF analysis showed increased cell count to 325 × 10^6^/L (normal range 0–4 ×10^6^/L) with mononuclear pleocytosis (99%), increased lactate level to 3,0 mmol/L (normal range<2,5 mmol/L), moderate increased total protein count to 2,6 g/L (normal range 0,15–0,50 g/L) and slightly lowered glucose level. The CSF/serum albumin ratio was increased to 26 (increased albumin in CSF to 1270 mg/mL, albumin in plasma 48 g/L), consistent with blood-brain barrier (BBB) dysfunction. The IgG-index was increased to 1,5 (cut-off 0,7), with increased IgG-antibody concentration in the CSF to 368 mg/L (normal range 0–34 mg/L). It was supplemented with isoelectric focusing that demonstrated the presence of multiple oligoclonal IgG bands in CSF that were not found in serum. Taken together, these findings were highly suggestive of intrathecal IgG antibody production due to neuroborreliosis, and treatment with intravenous ceftriaxone was initiated.

*Borrelia burgdorferi*-specific IgM and IgG antibodies were identified using Chemiluminescence Immunoassay (CLIA). Comparison of the concentration of *Borrelia burgdorferi*-specific antibodies in serum and CSF, showed a positive antibody-index, that is highly specific for Lyme neuroborreliosis. The polymerase chain reaction (PCR) protocols that tested for the *Borrelia* specific genes 16 S rRNA and OSP-A, who account for most of the *Borrelia* infections in Europe, were negative both in serum and CSF, but the sensitivity for PCR detection is low.

The CSF was also tested with FilmArray multiplex PCR Meningitis/Encephalitis panel, a protocol using a range of PCR assays for detection of 14 agents (Enterovirus, Herpes simplex virus 1 and 2, Varicella zoster virus, Cytomegalovirus, Human herpes virus 6, Human parechovirus, Neisseria meningitidis, Haemophilus influenzae, Streptococcus pneumoniae, Listeria monocytogenes, Streptococcus agalactiae, Escherichia coli, Cryptococcus neoformans/gattii), and all were negative.

Extensive testing of blood, respiratory secretions and CSF for other infectious agents were all negative, including Epstein-Barr virus (EBV), Cytomegalovirus (CMV), Toxoplasma gondii, Treponema pallidum, Brucella, Mycobacterium tuberculosis, Parainfluenza 1/2/3/4, Respiratory syncytial virus (RSV) A/B, Influenza A/AH1/B, Adenovirus, Human metapneumovirus (HMPV), Chlamydophila pneumoniae, Mycoplasma pneumoniae, Bordetella pertussis, SARS-CoV-2, Tick-borne encephalitis-virus (TBE) and JC polyomavirus (JCV). Human immunodeficiency virus (HIV) screen was negative and interferon gamma release assay (IGRA) test regarding *Mycobacterium tuberculosis* was also negative.

Autoimmune etiology was considered, but extensive serological tests gave normal or negative results, including normal erythrocyte sedimentation rate (SR), normal angiotensin-converting enzyme, negative screening for autoimmune vasculitis (negative antinuclear antibodies and antineutrophil cytoplasmic antibodies) and negative screening for autoimmune encephalitis (negative anti-NMDA-receptor, anti-AMPA-receptor 1 and 2, anti-GABA B receptor 1 and 2 IgG, anti-DPPX IgG). Anti-CASPR2 was positive, but the titer was low and considered unspecific and without clinical consequence.

The patient received 4 weeks of intravenous ceftriaxone 2 g/day, and she experienced immediately improvement in her general condition and both the headache and intermittent fever subsided. A follow-up assessment six weeks after initiation of antibiotic therapy showed further improvement of her symptoms although she still had some paresthesia and unsteady gait. The MRI findings in rhombencephalon had almost disappeared. Another scan seven months after initiation of therapy was completely normal, and she had further improvement of her symptoms.

## Discussion

Infections are one of the causes of rhombencephalitis, with *Listeria monocytogenes* as the most common agent followed by Enterovirus 71 and Herpes simplex virus (HSV) [13]. *Borrelia burgdoferi* is exceedingly rare. Incidence rates of CNS listerosis are 7,4 - 16 cases per million population [13], and rhombencephalitis are described in about 9% of these [20]. We did not detect *Listeria monocytogenes* in CFS or in blood cultures, and our patient was responding to ceftriaxone that would not have been the case if the patient had *Listeria* rhombencephalitis.

Other causes of rhombencephalitis include autoimmune diseases, with Multiple sclerosis (MS) and Behcet´s disease as the most common, and paraneoplastic syndromes [21]. Paraneoplastic syndromes causing rhombencephalitis have been associated with anti-Yo, anti-Tr, anti-Hu, anti-Ri, anti-Ma and anti-amphiphysin antibodies [13]. Our patient did not have other clinical or biochemical manifestations consistent with an autoimmune disease or malignancy, and treatment with ceftriaxone lead to clinical resolution. That would not have been the case with an autoimmune or malignant disease.

Clinical findings in rhombencephalitis depend on the underlying etiology and are highly variable. In general, the most common are cranial nerve palsies, which occurs in about 75% of patients, and cerebellar ataxia, especially if the cause is infection or a paraneoplastic syndrome [21]. Regardless of cause, patients with rhombencephalitis usually have CSF pleocytosis and increased total CSF protein. Brain MRI can be normal, but typical pathological findings are increased signal intensity in the rhombencephalon on T2-weighted and FLAIR MRI scans [13], as in this case report.

We present a patient that developed late stage neuroborreliosis with involvement of rhombencephalon. She presented with fever, weight loss and poor general condition, unspecific neurological symptoms, absence of the characteristic erythema migrans, normal non-contrast MRI of her brain about 5 months after onset of symptoms, and normal initial laboratory investigations. This demonstrates how challenging the diagnosis neuroborreliosis can be.

The patient developed significant increases in the tier of specific Borrelia IgG-antibodies in serum, and pathological findings in rhombencephalon was found on contrast enhanced MRI about 8 months after onset of symptoms. The CSF analysis revealed typical findings as seen in patients with neuroborreliosis [17], including increased CSF cell count, signs of BBB dysfunction and presence of *Borrelia burgdorferi*-specific IgM and IgG antibodies. Laboratory findings indicated intrathecal synthesis of specific antibodies against *Borrelia burgdorferi*, with positive specific antibody-index and presence of CSF oligoclonal IgG bands. Plasma and CSF samples were PCR negative, but the sensitivity is low, and it is important to notice that a negative PCR does not exclude the diagnosis [1]. Significant improvement of neurological symptoms and resolution of the MRI findings after proper treatment confirmed the diagnosis neuroborreliosis with rhombencephalitis.

The patient met all the criteria that The European Federation of Neurological Societies (EFNS) has established for diagnosis of acute definite Lyme neuroborreliosis, that are: (1) neurological symptoms suggestive of Lyme neuroborreliosis without other obvious reasons, (2) CSF pleocytosis and (3) intrathecal *Borrelia burgdorferi* antibody production [22]. American Academy of Neurology (AAN) have the following criteria for the diagnosis of neuroborreliosis: (1) possible exposure to *Ixodes* ticks in Lyme-endemic area, (2) one or more of the following: (a) erythema migrans, (b) histopathologic, microbiologic, or polymerase chain reaction proof of *B. burgdorferi* infection, (c) immunologic evidence of exposure to *B. burgdorferi*, (3) occurrence of a clinical disorder within the realm of those associated with Lyme disease, without other apparent cause [11]. Thus the patient fulfils both the European and American diagnostic criteria.

Clinicians should be cognizant that infection with *Borrelia burgdorferi* sensu lato can affect many organs, including the central nervous system. The neurological symptoms and findings can be nonspecific and partly depend on which parts of the nervous system that is affected [15]. No specific pattern on neuroimaging has been identified, and findings can also be normal, especially in early stage of the disease [12]. This may complicate and delay correct diagnosis, potentially leading to a worse outcome. If the clinical picture points in the direction of neuroborreliosis treatment is initiated as soon as possible while awaiting further test results.

## Conclusion

Rhombencephalitis refers to inflammatory diseases of the rhombencephalon that include the pons, cerebellum and medulla oblongata. *Borrelia Burgdorferi* sensu lato is an extremely rare cause yet important to recognize, as a good prognosis without neurological sequels is dependent on timely antibiotic treatment. We therefore recommend that neuroborreliosis should be considered among the differential diagnoses in patients where neurological symptoms and findings suggesting inflammation in the CNS, despite unusual or normal neuroimaging findings. It should especially be considered if the patient live in or have traveled to areas where ticks reside, or if history of possible erythema migrans.

## Funding

This research did not receive any specific grant from funding agencies in the public, commercial, or not-for-profit sectors.

## Consent

Written informed consent was obtained from the patient for publication of this case report and accompanying images. A copy of the written consent is available for review by the Editor-in-Chef of this journal on request.

## CRediT authorship contribution statement

**Hilde Svingen**: Conceptualization, Writing – original draft, Writing – review & editing. **Jon Orrem**: Writing – review & editing. **Arne Nørgaard Eskesen**: Conceptualization, Writing – review & editing.
